# Capturing Intracellular pH Dynamics by Coupling Its Molecular Mechanisms within a Fully Tractable Mathematical Model

**DOI:** 10.1371/journal.pone.0085449

**Published:** 2014-01-17

**Authors:** Yann Bouret, Médéric Argentina, Laurent Counillon

**Affiliations:** 1 Université Nice Sophia Antipolis, CNRS, LPMC, UMR 7336, Nice, France; 2 Université Nice Sophia Antipolis, CNRS, INLN, UMR 7335, Valbonne, France; 3 Institut Universitaire de France (IUF), Ministère de l’Enseignement Supérieur et de la Recherche Scientifique, Paris, France; 4 Université Nice Sophia Antipolis, CNRS, LP2M, FRE 3472, Nice, France; University of Calgary, Canada

## Abstract

We describe the construction of a fully tractable mathematical model for intracellular pH. This work is based on coupling the kinetic equations depicting the molecular mechanisms for pumps, transporters and chemical reactions, which determine this parameter in eukaryotic cells. Thus, our system also calculates the membrane potential and the cytosolic ionic composition. Such a model required the development of a novel algebraic method that couples differential equations for slow relaxation processes to steady-state equations for fast chemical reactions. Compared to classical heuristic approaches based on fitted curves and *ad hoc* constants, this yields significant improvements. This model is mathematically self-consistent and allows for the first time to establish analytical solutions for steady-state pH and a reduced differential equation for pH regulation. Because of its modular structure, it can integrate any additional mechanism that will directly or indirectly affect pH. In addition, it provides mathematical clarifications for widely observed biological phenomena such as overshooting in regulatory loops. Finally, instead of including a limited set of experimental results to fit our model, we show examples of numerical calculations that are extremely consistent with the wide body of intracellular pH experimental measurements gathered by different groups in many different cellular systems.

## Introduction

Distribution of charges within biological molecules is crucial, not only for reactivity and catalysis, but also as it determines their solubility, their particular folding, and dictates the spatio-temporal sequence of their interactions. In this context, the pH of the solution bathing these biological molecules is a key parameter, since its value determines the protonation of the acid-base groups that are especially abundant in macromolecular assemblies. Furthermore, as many enzymes and cellular regulators exhibit a strong pH dependency, the modification of the protonation of key residues can deeply impact their function. For these reasons, genomes necessarily contain pH-dependency information, which is expressed in the proteome [1]. The complete information for intracellular pH determination is a convoluted interplay between the abundance and the distribution of protonable groups in biological molecules, their pKa values and the expression, stability, kinetic and affinity parameters of the pH regulating systems. Accordingly, providing a fully tractable model for intracellular pH regulation is a challenging problem, and several studies have been aimed at building essentially heuristic models [2]–[5] for intracellular pH regulation.

The past decades have witnessed the detailed molecular characterization of the protagonists that regulate the concentrations of cellular acid-base equivalents, in term of both their kinetics and the affinities for their substrates [6], [7]. Significant efforts have also been invested to describe intracellular buffering mechanisms and proton diffusion in cells adequately [8], [9].

Based on this, we develop here a different, bottom-up approach at the interface between biology, physics, chemistry and mathematics. We construct a model that encompasses the individual molecular mechanisms for these regulators defined by their own kinetics and by their experimentally measured microscopic parameters. This requires the inclusion of the chemical reactions between the involved reactive species. This non-empirical process guarantees the construction of a physically coherent, fully integrated and tractable model (i) for cellular proton dynamics and (ii) for steady-state pH regulation.

In the present study, we choose to keep the system simple and modular by assuming that the cell surface and volume are fixed to their average values and by using the ubiquitous 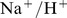 exchanger 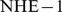 and 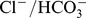 exchanger 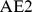 as the main transmembrane acid-base transporters. We also include the electrical gradient generated by the Na/K-ATPase across the membrane and the permeabilities associated to 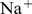, 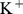 and 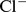 background currents measured in non-excitable cells. Therefore, our model computes the distribution of the other cationic and anionic species and their variations as a function of proton concentration.

These pumps and transporters show a very high sequence conservation within different mammalian species and possess very similar constants for their substrates. Based on this, we built our model using widely accepted values from the literature even if they had been measured from different mammalian species. We will further see that this is validated by our results, which show that pH regulation is very resilient against variations of those thermodynamic constants.

It is demonstrated that our model gives (i) a robust, experiment based prediction of the temporal evolution of the pH, (ii) a simple analytical value for its steady state, (iii) all the other ionic concentrations related to the proton regulation, (iv) and a reduced differential equation for describing the full pH balance.

This enables the testing of biologically-relevant situations whilst discriminating between critical parameters and rate limiting steps *versus* those factors that can be widely changed with virtually no effect on cellular homeostasis.

## Methods

### Datasets used for the Model

We report most of thermodynamical data, the common ionic environments, and the justification of the kinetic equations in the Datasets in File S1. In the following, we illustrate the specific behavior of the involved physical, chemical or biological components.

### Ionic Flows and Potential through the Membrane

**Figure 1:**
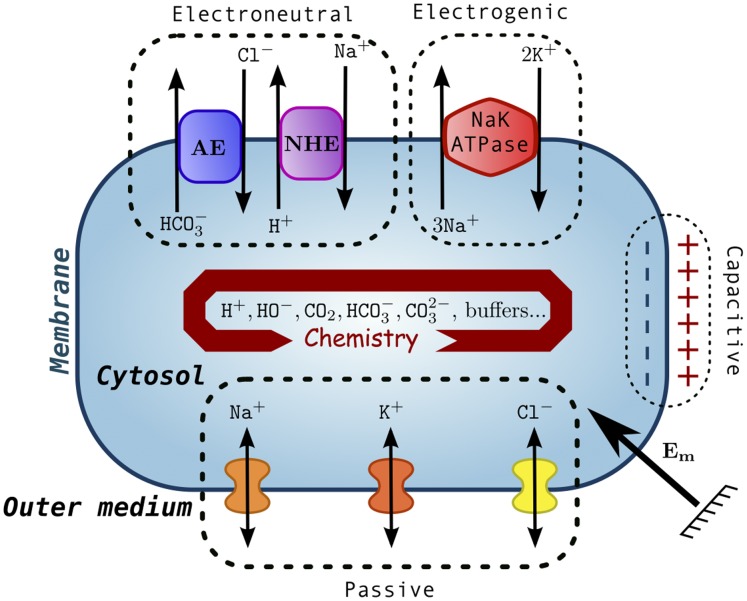
Cell model. The cell membrane acts as a capacitance, which is submitted to the membrane potential 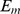. Three blocks exchange ions between the cytosol and the outer medium: they induce electroneutral transports (with the 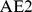 and 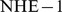 exchangers), electrogenic currents via the Na/K-ATPase, and passive ion channels (for 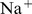, 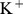, and 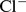 ). The chemical reactions are assumed to take place within an homogenous cytosol.

Let us depict the cellular model represented in Figure 1 mathematically. We assume that the cell geometry is fixed by neglecting that water flows through the membrane. The charge balance is controlled by passive, electroneutral, electrogenic flows and capacitive currents that are described as follows.

#### Passive flows

If 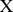 represents a chemical species in Figure 1, with an inner concentration 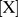 and an outer concentration 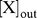, then it flows out of the cell through the membrane surface 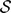 due to a permeability 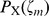. Here, 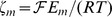 represents the reduced electric potential where 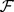 is the Faraday constant, 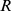 is the molar gas constant, 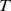 is the absolute temperature, and 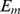 is the electric potential difference between the cytosol and the outer medium. The Goldman-Hodgkin-Katz flux equation [10] provides the outward molar flux 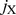 as
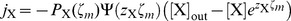
(1)with 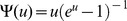 and 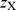 is the algebraic charge of 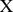. The associated passive outward electric flux is 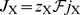 and the whole cell passive outward electric current is 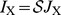. We can simply convert the flux into an intake molar rate for a given cell volume 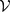 as



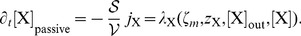
(2)For the cellular system used in the electrophysiological measurements we recorded significant currents only for 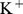, 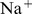 and 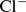 (CCL39 cells, see Figure S1 in File S1). This allows the determination of the corresponding permeabilities. Any other species can be taken into account if other cells are considered, and if values are available or measurable.

#### Electroneutral transports

The electroneutral 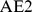 exchanger keeps 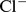 ion concentration above its Nernst potential [11], and thus is assumed to work in the forward direction. 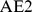 is then operating with a Hill mechanism [12], inducing a whole cell exchange rate
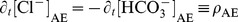
(3)with 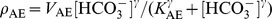 and where 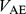 is the cellular maximal 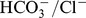 exchange rate, and where 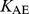 is the bicarbonate affinity. Unless indicated, we will use a Michaelis-Menten behavior, namely 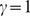, and 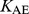 is about 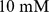.

We use the established mechanism [13] of the 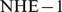 exchanger that results in the whole cell exchange rate
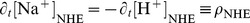
(4)with 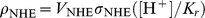 and where 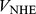 is the cellular maximum 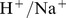 exchange rate, 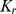 is about 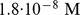 and



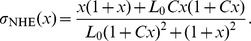
with 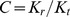, 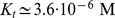 and 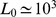.

Any other electroneutral transporter could be similarly described and therefore inserted into the model.

#### Electrogenic currents

We restrict ourselves to the sodium-potassium pump that exchanges three inner 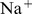 with two outer 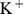 according to
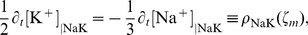
(5)where 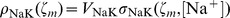. 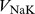 is the cellular maximum 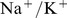 exchange rate, and we combined the experimental data found in published studies [14], [15] to estimate (see Datasets in File S1)



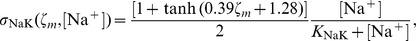
with 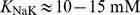.

#### Electric potential evolution

The cytosol and the outer medium must remain globally electroneutral. Conversely, charge accumulation polarizes the membrane due to its surface capacitance 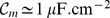 (see Materials and Methods in File S1). The total capacitance of cell membrane is 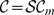.

We take into account both the passive and electrogenic actors, respectively defined by equations (1) and (5), which are involved in the electric potential regulation. This results in
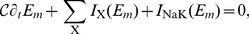
(6)with the electric conversion 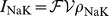.

### Chemical Physiology

So far in our modeling, the species passing through the membrane are 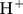, 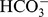, 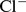, 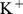 and 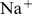. Obviously, the first two of them are directly involved in the set of protic reactions that govern the 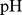. Since the physiological range of 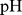 lies around 7, we must monitor in our analysis the self-ionization of water
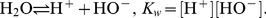
(7)


Then, we include the three components of the carbonated system. The partial pressure 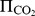 of carbon dioxyde equilibrates with aqueous 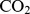 (according to the Henry law), which undergoes two consecutive dissociations. Those reactions and their equilibrium constants are summarized below:
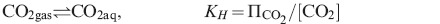
(8)

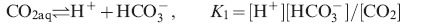
(9)

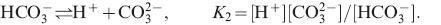
(10)


Since the dissolved 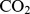 is very unstable in water, and especially in presence of physiological carbonic anhydrase [16], we shall directly merge the equilibria (8) and (9) to get rid of 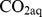 and obtain an equivalent equilibrium:
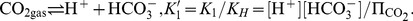
(11)


Finally, we model the protic behavior of all the other species within the cytosol by a single equivalent buffer that we name 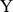, acting as
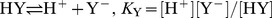
(12)with 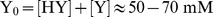 (see [8] for details). Additionally, we do not consider the diffusive effects within the cell, by assuming homogeneous ionic concentrations. Similarly, the outer media is considered as an infinite bulk with constant physiological concentrations of the different entities under consideration.

## Results

### General Theoretical Results

#### Self-consistency and modularity

To be self-consistent, our model must ensure that each ion and the membrane potential are sufficiently maintained by physico-chemical processes (enzymatic transport or chemical reaction) in order to avoid some non-physical accumulation or discrepancy such as negative concentration. Accordingly, we propose a model for a generic cell, restricted to the previous components (transporters, ionic permeabilities and chemical reactions) which fulfills these criteria. Noticeably, any other effector that acts redundantly for pH regulation or is expressed in specialized cells can be implemented, provided that the above self-consistency is preserved. As an example, we will show further how to handle the lactate/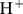 production and transport. The same methodology applies to other mechanisms (such as 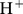-ATPase or 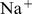-coupled-bicarbonate transporters) and any additional chemical reaction.

#### Full formal system dynamics

For any reaction indexed by 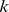, we will note 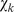 its associated rate, which is the derivative of its chemical extent with respect to time. For each chemical species, the concentration temporal derivative is the appropriate summation of the chemical molar rates, the exchanger transport rates and the passive intake molar rates. The full system is straightforwardly
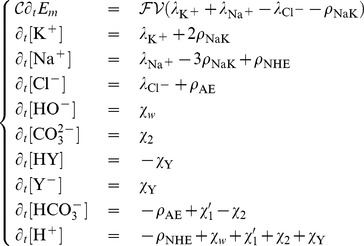
(13)


where 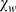, 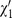, 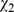, and 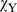 are respectively the molar rate of the water ionization (7), the direct formation of 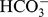 (11), the dissociation of 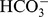 (10), and the deprotonation of the equivalent buffer (12). The first equation of the above system is rewritten from relation (6).

The main characteristic of protic reactions in water is that they have very short relaxation times, from a few microseconds for water [17] to a few milliseconds for 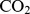 with carbonic anhydrase [16] or without carbonic anhydrase [18]. Since the transmembrane exchanges of protic species (through 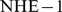 or 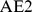) are expected to have characteristic times much larger, we can consequently make the assumption that each protic equilibrium is in fact a fast pre-equilibrium. It follows that each involved reaction quotient always matches its corresponding thermodynamical reaction constant: this can be seen as a set of constraints applied to the chemical composition of the aqueous solution. Consequently, if we want to impose a perturbation of this composition then those pre-equilibria shall instantaneously produce the mandatory chemical extents which ensure that the final composition respects the chemical constraints. Accordingly, the thermodynamical knowledge of the equilibria (7) to (12) is sufficient to solve the kinetic equations (13) in this particular biological context.

In the following, we detail the treatment of the protic reactions rates within the equations (13). We show (see Methods in File S1) how to derive a set of reduced differential equations for the 5 dynamic variables 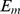, 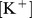, 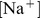, 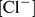 and 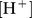, within this pre-equilibria approximation.

#### Steady state characterization

The steady-state values, which we note withan asterix, are obtained by setting to zero the temporal evolution in the differential system (13) leading to
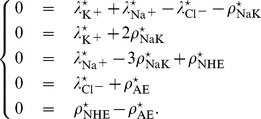
(14)


This system is under-determined since the sum of the first equation and of the last two ones minus the second and the third one is zero: this is expected since the evolution of 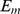 is the exact conservation of the global electric charge. In our model, the latter decomposes into an intrinsic charge 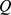 of all the considered components and an excess charge 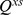 of all the other “spectator” species (proteins, other ions…), leading to 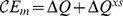, where 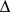 means the difference between the cytosol and the outer medium values. It is the integrated form of equation (6). As a consequence, the initial condition of the differential system (13) gives 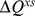.

In order to determine steady-state values, we first obtain the electric equation 
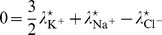
 which is here equivalent to
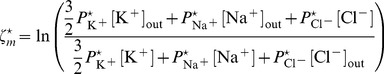
(15)since we only deal with monovalent ions. The relation (15) is the Goldman-Hodgkin-Katz potential equation with voltage-dependent permeabilities and a potential explicitly regulated by Na/K-ATPase.

With 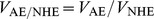 (see equations (3) and (4)) and 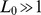, the last equation of the system (14) can be expressed as a function of 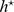 and it reduces to a simple polynomial
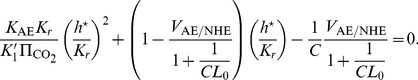
(16)


**Figure 2:**
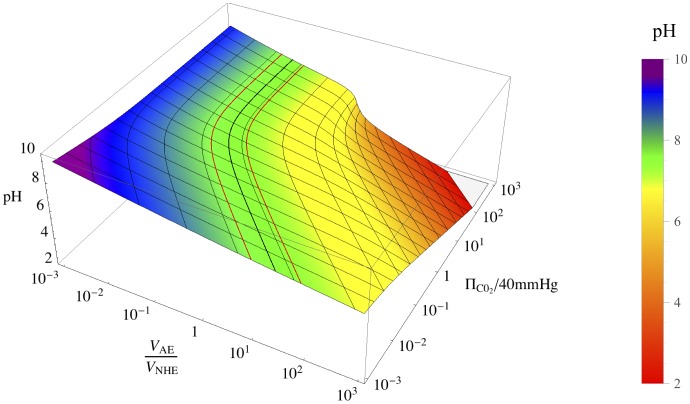
Three dimensional representation of steady-state pH. pH is drawn as a function of the two controlling parameters 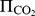 and 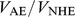. The flattest area of this surface stretches over 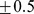 units around physiological pH (7.2, in black). This corresponds to values that can be reached by cellular systems (red boundaries).

This analytical relation yields the steady-state pH as a function of 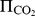 and 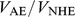, since 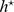 is the positive root of equation (16). The Figure 2 shows how the exact 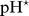 evolves when those parameters are changed and where the acceptable physiological limits stand. In particular, for an intracellular 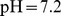 and 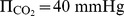 our model predicts 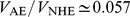: this transport ratio matches well the experimental maximal rates of this transporters in different systems [19]–[21]. An interesting feature of our model is the prediction of missing parameters (kinetic and/or thermodynamic) based on the knowledge of steady-state physiological values (see Results in File S1). For instance, a unique 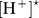 is computed from a given 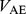, 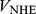 and 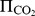. Conversely, the ratio 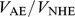 can be read on Figure 2 from the experimental measure of 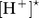 and 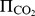.

### Asymptotic Kinetics Framework

#### General philosophy

The set of differential equations (13) defines a multiple-scale system (in both time and in concentrations), since it combines slow chemical rates with fast relaxing protic reactions. In either muliple-scale analysis [22] and normal forms in central manifolds [23], the slow dynamics are assessed around a stationary point. However, in the case used herein, the slow dynamics evolve on manifolds generated by the laws of mass action (corresponding to each protic pre-equilibrium established at its thermodynamical constant) and represent the only valid compositions of the system. To the the best of our knowledge, this is the first time that a way to compute the constrained evolution of the all the involved concentrations has been exposed.

#### Chemical system description

If we assume that we have 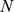 chemical reactions coupling 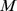 species 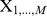, then the relevant reactions may be written in a generic form, employing the *algebraic* stoichiometric coefficients 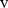 :
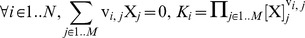
(17)where 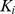 is the equilibrium constant of the 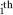 reaction. We also assume that 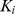 is temporally dependent, so as to reflect the possible variations of the external conditions (such as imposed changes in partial pressures).

#### Fast pre-equilibria consequences

We now assume that those 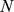 reactions represent fast pre-equilibria. In other words, we suppose that the relaxation time of each reaction is infinitely small. Accordingly, we define the vector 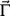 by its coordinates 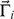 such that
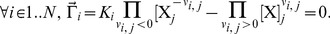
(18)


Thus, the only permissible evolutions must be satisfied at particular time 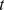 and for any set of concentrations 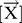 through the relationships:
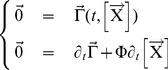
(19)where 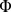 is the Jacobian matrix of 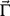 with respect to 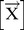.

#### Response to perturbations

If we perform a small modification 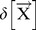 during 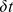 on this system, all the reactions evolve to preserve 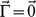. The resulting individual chemical extents 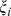 of each reaction form the vector 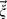. We then obtain a modified perturbation
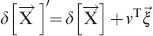
(20)and this in turn *must obey*



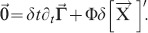
(21)The instantaneous chemical extent is readily computed by
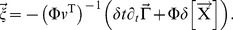
(22)


We can show that the 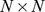 matrix 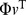 is invertible for any admissible set of concentrations. This purely algebraic property results from the convexity of the free enthalpies of reactions from which the expressions of 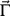 and 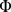 are derived, but this purely mathematical demonstration is far beyond the scope of this article.

#### Generic asymptotic kinetics

Finally, noting that 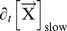 contains global information regarding the “slow”-changing variations of all the chemical species, the overall chemical evolution of the system is deduced from (22) by setting 
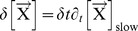
. Thus, asymptotic kinetics can be written as
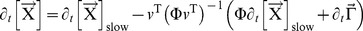
(23)thereby illustrating how the fast chemical reactions are damping the slow variations.

#### Numerical integration

A modular C++ program (available upon request) was designed and exactly encodes the biological effectors, the membrane potential and the chemistry into a system of algebraically coupled numerical differential equations. The integration step was performed by an adaptive Dormand-Prince method with a fractional tolerance of 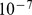.

### Reduced Model for pH Dynamics

#### Evaluation

If it is assumed that all the protic reactions are rapid pre-equilibria (see above), then we can derive the proton generation rate by using the previous mathematical formalism. This formalism provides an algebraic manner to decouple all the protic reactions from the catalytic ones explicitly. Implicitly however, all protic dynamics may be deduced from the evolution of 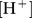 which has unfortunately no simple expression.

#### Simplified pH dynamics

For a given cell in physiological conditions (for which the internal steady pH is around 7.2) we can neglect the presence of 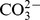 and obtain a slightly simplified rate (see Methods in File S1):
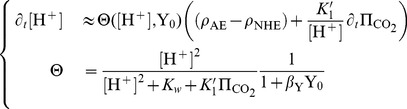
(24)with a numerically derived value 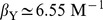, and for a total buffer concentration 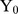. Here, 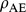 and 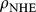 depend only on the pH and on 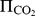, so that one can simulate the pH with only *one* differential equation.

#### Role of the buffer

The steady-state 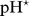 is readily recovered from equation (24). The factor 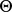 emphasizes the preponderant role of the chemical couplings pertaining to the evolution of the intracellular pH. Indeed, we always have 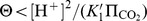, so that for the steady state of a “normal” cell (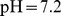, 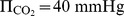), we estimate 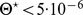. The protons dynamics (hence of all the protic species) are sharply damped by those chemical couplings. As expected, the buffer causes these dynamics to be even further reduced through the term 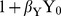 in the 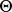 denominator, up to 30% for a 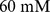 buffer concentration.

#### Natural overshoot

Interestingly, our calculations predict that a vanishing physiological protic perturbation will systematically produce a pH overshoot around its steady state. Such phenomena are well known experimentally [24], [25]. The exact mathematical demonstration of this phenomenon (see Methods in File S1), is valid for acidification or alkalinization, and can be applied to model other overshoots observed in different physiological regulations.

To explain this in a non-mathematical way, we may perform the following thought experiment. Let us assume that a weak protonated base 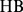 enters the cell at its steady state. The excess of protons produced by the dissociation 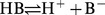 is continuously pumped out of the cell by the regulating enzymes. As a consequence, if the cell removes 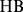 from its cytosol, then some protons will not neutralize the remaining 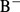. Accordingly, the initial pH is reached earlier than expected: the further removal of 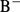 straightforwardly creates an unexpected depletion of protons (basic environment) before returning to the initial situation. This describes an overshoot mechanism.

### Steady-state pH: Role of Enzymatic Constants

We have investigated the changes in the steady-state 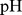 resulting from covalent or non-covalent modification of the transporters through intracellular signaling cascades, and the effects of allosteric activators or inhibitors or mutations that affect the transporters parameters. Unless stated, we model these effects assuming that a modification is specific and affects only one thermodynamical enzymatic constant without changing the others, while we keep the kinetic ratio 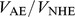 and 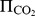 to their usual levels.

**Figure 3:**
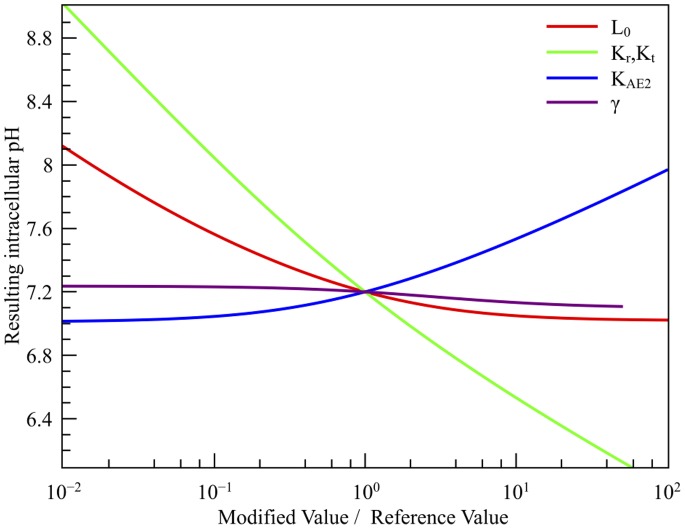
pH modification by changes in biochemical constants. The reference values are 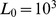, 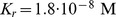, 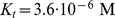, 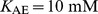 and 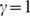. For 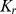 and 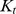 modification, the ratio 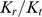 is held constant (see text for the explanation).

For 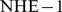, it has been shown that within the Monod-Wyman-Changeux framework [26], the allosteric constant 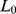 is modified by various stimuli such as growth factor stimulation or changes in membrane composition and tension [13], [21]. This raises the question whether 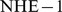 cooperativity for proton is intrinsically important for pH regulation itself and other cellular functions. Accordingly, Figure 3 depicts the results of our computations of the resulting pH following a modification of 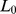. Interestingly, it has been shown widely that the activation of 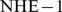 by the above-mentioned stimuli [27] decreases 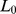 by one order of magnitude [13], and results in a pH increase of 0.2 to 0.3 units. Our model yields a pH increase of about 0.3 units for 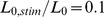, which is in very good agreement with the actual experimental data. We are also able to hypothesize that in some cases 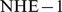 might be regulated by altering its microscopic affinities for protons. To investigate this, we changed the 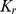 and 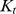 constants of 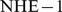 and kept the 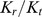 ratio constant, as these two affinities correspond to the same site in different conformations. Our model predicts that any important change in these microscopic affinities would produce a large pH shift, as shown on Figure 3.

For 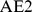, we investigated either the effect of a variation in the affinity 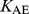 or in the Hill exponent (see Figure 3). We observe a less drastic change than those resulting from a 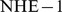 modification. This makes sense from a physiological point of view: due to membrane potential and metabolic activity, cells constantly have to compensate for intracellular acidification rather than for alkalinization.

To summarize, we show that pH regulation is robust for two main reasons. Firstly, changes in the thermodynamic constants of pH regulation systems, that *in vivo* could arise from mutations or from interspecific variations, induce minute modifications of the steady-state pH. Secondly, pH can relax back to its physiological value, because changes in constants are very easily overcome by slight modifications of the maximal rates of the transporters, *i.e.* the amount of transporters expressed at the plasma membrane. This *a posteriori* validate the hypotheses and choices described in the introduction.

### Characteristic Time Scales in pH Regulation

In order to investigate the dynamics, we integrated the differential system (13) as described previously. We choose to approximate the average fibroblaste shape using a prolate spheroid model of length 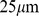 and a diameter of 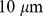, leading to a surface 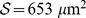 and a volume 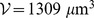.

We found an anionic charge excess of 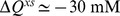 for this configuration, which mainly corresponds (i) to the excess of negative charges found on the surface of intracellular proteins and (ii) to the bulk of negative charges provided by the first dissociation of phosphate groups [8].

#### Relaxation times around the steady-state values

We performed the linear stability analysis of the differential system (13) which also provides the relaxation constants of the independent variables. We deduced the raw and typical relaxation time constants for our cell model by setting the equivalent buffer concentration to zero. Firstly, we obtain a 3 ms characteristic time that predominantly corresponds to the relaxation of 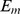: obviously the membrane potential adjusts itself very quickly to a change in the ionic composition, but since it does not produce chemical species *per se*, it does not influence the chemical rates.

Secondly, we have two similar time constants representing the relaxation of a perturbation of all the ions within 8 and 15 minutes.

Finally, for a fixed 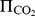, the perturbed concentration 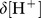 dynamics obeys to
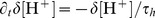
(25)with 
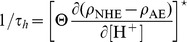
. Since the relation (24) provides 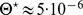, the proton relaxation time 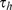 reaches about 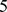 minutes, which is consistent with the experimental observations from a plethora or reports [24], [28], [29].

### Illustration on Pathophysiological Situations

#### Forced acidosis: 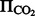 and intracellular buffer

**Figure 4:**
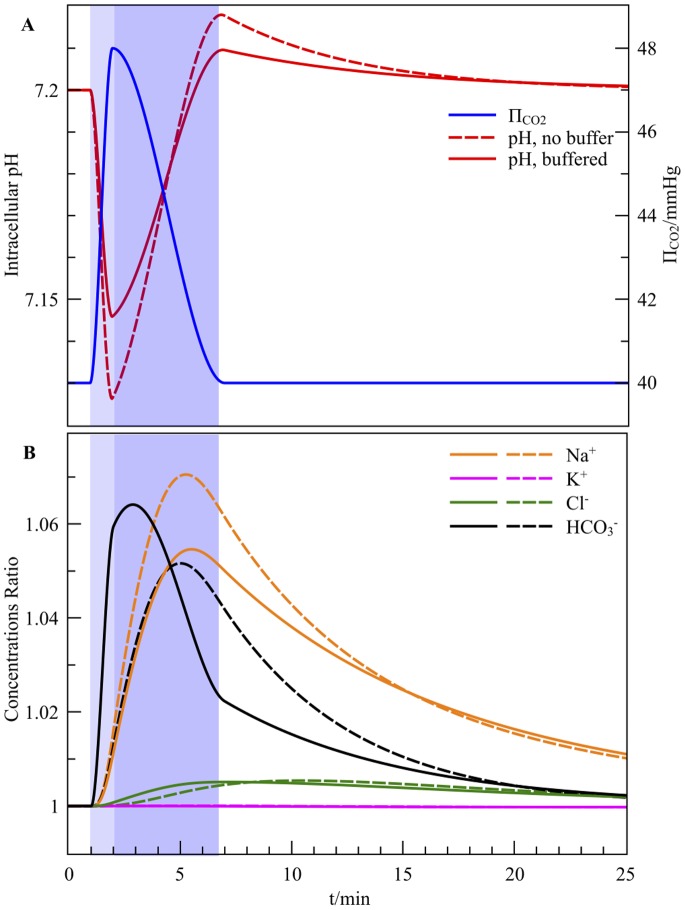
Forced acidosis by a simulated 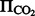 spike. The rise and the decrease of 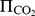 are highlighted by blue areas. (A) The expected pH overshoot takes place with or without the presence of a 60 mM physiological buffer (dashed lines). (B) The ionic ratios relative to the initial values are reported as well.

We simulated an artificial increase of 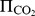 of 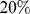 during 1 minute (from 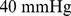 to 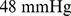) followed by a return to the normal within 5 minutes. The resulting pH dynamics with and without the equivalent buffer 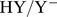 are presented in Figure 4A. As shown in Figure 4B, the 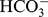 excess increases the 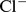 intake, and the acidification increases the 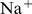 intake, while the 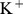 level is remarkly stable as expected. The net ionic currents produce a concurrent tiny 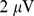 depolarization. We note that, *via* the chemical couplings, the aprotic species dynamics are also dampened by the presence of the buffer. As expected, the different timescales are also respected once the perturbation is over and all the concentrations are relaxing towards their steady-state value: the pH needs only a few minutes to recover its physiological level, while the other ions rather require tens of minutes to reach their final balance. The numerical simulation also points out the predicted overshoot with and without buffering.

If 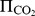 is held to at its maximum increase, then the pH converges to a new value that can be deduced from Figure 2. In such a case, the pH curve is similar to Figure 4A except that it converges monotonously towards almost 7.18 after the initial decrease, and no overshoot occurs. At the same time, the other ions find a different balance. The results of this specific 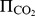 jump are shown on Figure S2A&B in File S1.

#### High-flow lactic ischemia

**Figure 5:**
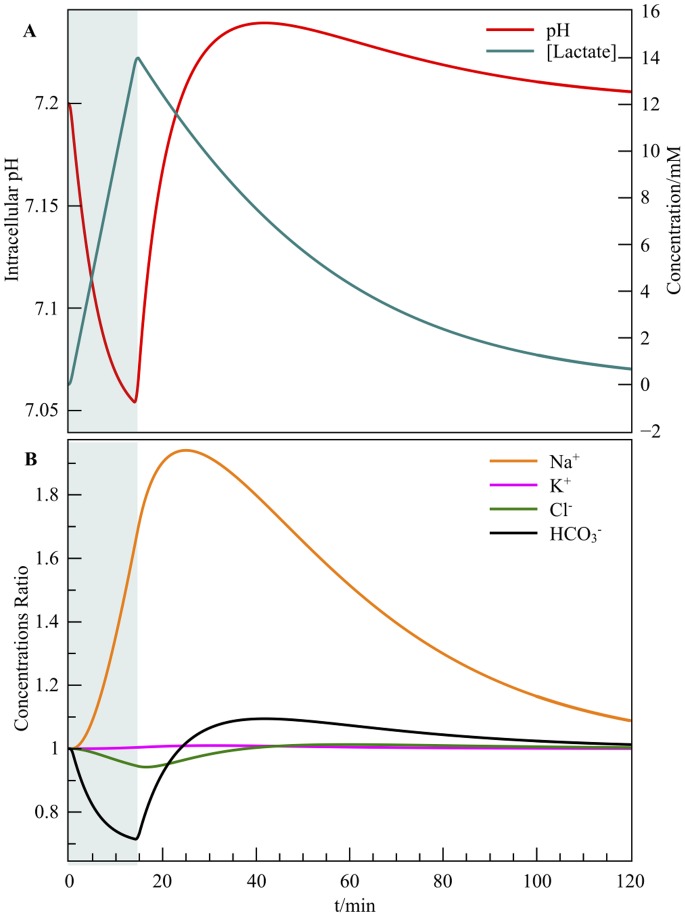
Simulated ischemia. Lactate production occurs with a constant 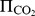, a 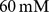 buffer, and with transporters working at their nominal level. The lactate accumulation is highlighted by a green area. (A) The predicted pH overshoot takes place during the lactate removal by the monocarboxylate transporters. (B) The corresponding ionic ratios relative to the initial values.

Here we show how to expand the model in order to probe the consequences of a slight hypoxia without ATP depletion. We model it with a lactic acid production, while we hold the enzymatic constants and 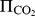 to their normal values. Since our model is modular, we first consider the dissociation of the lactic acid 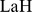, namely 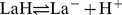 with 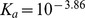. The cell removes lactates and their accompanying protons (1:1) through monocarboxylate transporters (MCT) [30], [31]. The latters follow a Michaelian law defined by 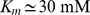 and by an observed maximum rate 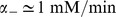 [32]. The global lactic acid production is around 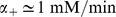 in an hypoxic skeletal muscle [33]. Consequently we simply have (i) to append
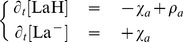
(26)where 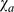 is the molar rate of the lactic acid dissociation and (ii) to include 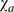 to 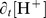 within the differential system (13). Here we impose
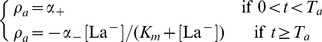
(27)where 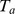 is the ischemia duration. The resulting pH is shown in Figure 5A.

Consequently, an extra term appears in the overall protonic rate:
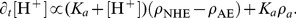
(28)


The acidification of the cytosol produces a massive 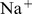 overload (see Figure 5B), which is experimentally observed [28] and corresponds to a stimulated action of 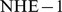. For a fixed 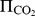, the fall of 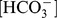 during the lactate production decreases the chloride intake, as shown in Figure 5B. The net ionic currents induce a small 200 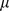
V hyperpolarization over the simulation. As demonstrated in the Methods of File S1, the structure of equation (28) leads to an expected pH overshoot, which occurs after 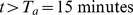. The fast regulating couple formed by 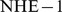 and 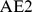 allows the pH to follow the rate limiting lactate expulsion.

## Discussion

### Adequacy with Experimental Data

As previously stated, the main purpose of this study was to build a mathematical depiction of intracellular pH regulation and investigate whether it had analytical solutions and produced biologically relevant simulations. This last section intends to further challenge our study by confronting real experimental data. To avoid potential biases, we decided against the use of our own data and instead to choose one of the pioneer experiments within the large body of published intracellular pH measurements generated by independent groups in the last four decades. Namely, we use here experimental recordings performed in one of the chief studies on intracellular pH regulation published by Roos and Boron in 1981 [24]. In this study (figure 5A of the original article), a *Helix* neuron was submitted to a 10 minutes 5% 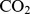 pulse. Its pH dropped from 7.35 to 6.85 and returned to normal after the pulse, with a noticeable overshoot. Details of the calculation and graphical results produced by our simulation are given in Materials and Figure S3A in File S1. Taken together, they show that only very minimal modifications of the constants of the system, well within differences found between different cell lines such as fibroblasts and neurons, have to be applied to converge to the resting intracellular pH measured in experimental conditions and that very satisfactory matches are obtained between calculated and experimental values for 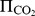 and intracellular pH.

### Main Outcome

This study describes the first fully coupled and self-consistent mathematical system for intracellular pH regulation. For this, we constructed a minimal system that is uniquely based on the kinetic, electric and chemical equations describing the molecular processes pertaining to intracellular pH. This strategy is very different from classical heuristic methods used to model biological processes, that are mostly built on phenomenological equations deduced from fitted curves. It also avoids the introduction of *ad hoc* fluxes and/or constants to ensure the convergence of the numerical simulations with experimental data. Importantly, the present approach allows analytical processing. It shows, for the first time, that the dynamics of pH can be described by a reduced differential equation, and that steady-state intracellular pH values are in fact analytical solutions. Besides, despite the formal complexity provided by the large body of equations used here, the calculated numerical values of pH, ionic concentrations and membrane potential converge towards physiological values, with time evolutions that are very reminiscent of experimental behaviors. The last remarkable finding is the demonstration that any additional phenomenon that directly or indirectly impacts pH can be mathematically included without violating our model, provided that its equation is not ill defined. At this step, it is important to notice that here, we focused on the construction of a model restricted to intracellular conditions in a single, isolated and homogenous cell. Because our system is fully modular it will enable future refinements. In particular, future developments will include cell shape and mechanics, extracellular physical and chemical parameters as well as diffusive transport.

## Supporting Information

File S1 **Supporting information files. Datasets.** Thermodynamical Constants, Cell Physicochemical, Environment Kinetic Parameters, Na/K-ATPase currents: voltage and sodium dependencies. **Electrophysiological studies of CCL39 cells.** Materials and Methods, Results. **Methods** **.** Obtaining an analytical expression of the pH dynamics, Natural Overshoot. **Results.** Simulation, Computing enzymatic constants from steady state values, Experimental Adequacy.
(PDF)
