# Supplementary material for: Capturing Intracellular pH Dynamics by Coupling Its Molecular Mechanisms within a Fully Tractable Mathematical Model
Source: PLoS One. 2014 Jan 17;9(1):e85449. doi: 10.1371/journal.pone.0085449 (PMC3894979; doi:10.1371/journal.pone.0085449)
Supplement: File S1 — Supporting information files. Datasets. Thermodynamical Constants, Cell Physicochemical, Environment Kinetic Parameters, Na/K-ATPase currents: voltage and sodium dependencies. Electrophysiological studies of CCL39 cells. Materials and Methods, Results. Methods . Obtaining an analytical expression of the pH dynamics, Natural Overshoot. Results. Simulation, Computing enzymatic constants from steady state values, Experimental Adequacy. (PDF) [file pone.0085449.s001.pdf]

Capturing intracellular pH dynamics by coupling its molecular mechanisms within a fully tractable mathematical model

Yann Bouret[1], Médéric Argentina[2,3], Laurent Counillon[4,*]
1 Univ. Nice Sophia Antipolis, CNRS, LPMC, UMR 7336, 06100 Nice, France
2 Univ. Nice Sophia Antipolis, CNRS, INLN, UMR 7335, 06560 Valbonne, France
3 Institut Universitaire de France (IUF), Ministère de l'Enseignement Supérieur et de la Recherche Scientifique
4 Univ. Nice Sophia Antipolis, CNRS, LP2M, FRE 3472, 06100 Nice, France
⋆ E-mail: laurent.counillon@unice.fr

Datasets

Thermodynamical constants

The thermodynamic constants used in our study are provided in Table S1, from Physics and Chemistry Handbooks.

Constant	Value
K_w	10^{-14}
K_H	29.41 atm.mol.L^{-1}
K_1	$4.45 \cdot 10^{-7}$
K_2	$4.69 \cdot 10^{-11}$
K_Y	$10^{-6.2}$

Table S1. Thermodynamic constants used for computations. For a temperature of 298 K.

For simplicity, we neglect the effects of temperature variations since it would shift their values by a few percents only.

Cell physicochemical environment

We use the typical ionic atmospheres and membrane potential for intra/extra media [1], similar to our experiments, and reported in Table S2.

Parameter	Intracellular Value	Extracellular Value
$pH^\star$	7.2	7.4
$E_m^\star$	−60 mV	–
$[K^+]^\star$	140 mM	4 mM
$[Na^+]^\star$	10 mM	140 mM
$[Cl^-]^\star$	20 mM	100 mM

Table S2. Steady-state ionic atmospheres. These values correspond to our electrophysiological experiments.

Kinetic parameters

The constants for AE2, Na/K-ATPase, and monocarboxylates transporters are obtained from the literature cited in the text.

The equation used in this study for the Na^+/H^+ exchanger NHE–1 is derived from [2]. Briefly, initital rates of transport for NHE–1 were measured in cells clamped at various intracellular pH values using fast kinetics of $^{22}Na^+$ and/or Li^+ uptake. NHE–1 initial rates were calculated as the cariporide (10 μM)-sensitive Na^+ or Li^+ accumulation per well divided by the corresponding protein concentration. The maximum rate for NHE–1 was measured as the rate of Na^+/H^+ exchange at saturating external cation concentration for an intracellular pH of 5.2, per mg of cellular proteins. Data were compiled using Microsoft Excel software and fitted using the Sigmaplot 2001 program (Jandel), the experimental points corresponding to the compilation of at least five independent experiments, with each experimental point determined at least in duplicate.

Na/K-ATPase currents: voltage and sodium dependencies

We used the litterature data (see below) to model the ionic currents delivered by Na/K-ATPase. Indeed, in our model, Na/K-ATPase is a current generator that helps regulating the membrane potential E_m by replacing 3 internal $\mathtt{Na}^+$ by 2 external $\mathtt{K}^+$. Accordingly, since the external concentrations are fixed, we assessed the variations of this current with respect to both E_m and the internal $[\mathtt{Na}^+]$.
A first work [3] deeply investigated the Na/K-ATPase current $I_{\mathtt{NaK}}$ with respect to the membrane potential. We fitted the accompanying figures of this work with the reduced potential $\zeta_m = \mathcal{F}E_m/(RT)$ to obtain an enzymatic sigmoidal shape

$$I_{\mathtt{NaK}} \propto 1 + \tanh\left((0.39 \pm 0.02)\,\zeta_m + (1.28 \pm 0.03)\right) \tag{1}$$

where the standard errors are indicated and with a correlation coefficient is greater than 0.999. Since Na/K-ATPase is an ubiquitous protein, we assume that this expression is verified for any cell type.
Obviously, we need to explicit the variation of $I_{\mathtt{NaK}}$ with $[\mathtt{Na}^+]$ since a $\mathtt{Na}^+$ depletion will stop the activity of Na/K-ATPase. We directly use the thermodynamical results of a second work [4] which states a Michaelian-like activity with a sodium affinity constant $K_{\mathtt{NaK}} \simeq 10 - 15$mM.
As as consequence, we combined those two results to produce a voltage-dependant Michaelis-Menten kinetics as

$$\partial_t\,[\mathtt{Na}^+] = -3 \times V_{\mathtt{NaK}} \frac{1 + \tanh\left(0.39\zeta_m + 1.28\right)}{2} \frac{[\mathtt{Na}^+]}{K_{\mathtt{NaK}} + [\mathtt{Na}^+]} \tag{2}$$

where $V_{\mathtt{NaK}}$ is the catalytic rate for the resting membrane potential.

Electrophysiological studies of CCL39 cells

Materials and Methods

Whole CCL39 cell currents recorded at room temperature were sampled at 2.5 kHz and filtered at 1 kHz. The cells were held at -50 mV, and 400 ms pulses varying from -100 to +100 mV were applied in 20 mV increments.
$\mathtt{Cl}^-$ currents recordings were performed using a pipette solution containing (in mM): 145 NMDGCl, 10 HEPES (pH 7.4), 5 MgATP, 5 EGTA (Π_{os}= 290 mOsm/kg H_2O). The bath solution contained (in mM) : 140 NMDGCl, 10 HEPES (pH 7.4), 5 MgATP, 5 EGTA and 50 Mannitol (Π_{os}= 320 mOsm/kg H_2O).
To study $\mathtt{K}^+$, conductances pipette solution contained (in mM): 140 K-gluconate, 10 HEPES (pH 7.4), 10 EGTA, 1 $CaCl_2$ (free calcium concentration of 10 nM) and 1 MgATP (Π_{os}= 290 mOsm/kg H_2O). The bath solutions contained (in mM): 145 NaCl or 145 K-gluconate, 10 HEPES (pH 7.4), 5 glucose, 1 $CaCl_2$, 1 $MgCl_2$ and mannitol (Π_{os}= 320-330 mOsm/kg H_2O).
$\mathtt{Na}^+$ conductances recordings were performed using pipette solution containing (in mM) : 145 Na-gluconate, 10 HEPES, 5 EGTA (Π_{os}= 300 mOsm/kg H_2O, pH 7.4). The bath solution contained (in mM) 145 Na-gluconate, 10 HEPES, 1 $MgCl_2$, 1 $CaCl_2$ (Π_{os}= 300 mOsm/kg H_2O, pH 7.4).

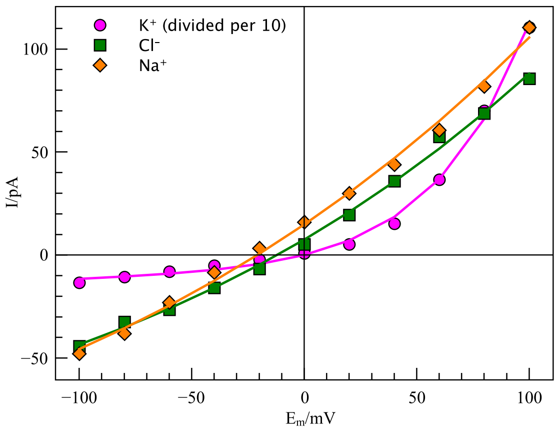

Figure S1. I/V curves for $\mathtt{K}^+$, $\mathtt{Na}^+$ and $\mathtt{Cl}^-$ in CCL39 cells. The symbols •, ▪ and ◆ represent respectively the $\mathtt{K}^+$, $\mathtt{Na}^+$ and $\mathtt{Cl}^-$ currents. The $\mathtt{K}^+$ currents are divided by 10 to allow its presentation on the same scales as the $\mathtt{Cl}^-$ and $\mathtt{Na}^+$ currents. The solid curves are the fit results from the permeabilities.

Results

The resulting experimental currents (averaged over 8 trials for each ion) are reported on Figure S1, where the $\mathtt{K}^+$ currents are divided per ten. Cell capacitance was typically in the range of 5 to 10 pF, leading to a surface capacitance of about 1μF.cm^{-2} (in good agreement with the literature data [5]).
We estimate the ionic permeabilities as follow. Let $\mathtt{X}$ be a chemical species with a cytosolic concentration $[\mathtt{X}]$, an outer concentration $[\mathtt{X}]_{\text{out}}$ and an algebraic charge $z_{\mathtt{X}}$. If we consider that $\mathtt{X}$ flows through the surface $\mathcal{S}$ of the cell membrane which is polarized by an electric field E_m, or $\zeta_m = \mathcal{F}E_m/(RT)$, and with a permeability $P_{\mathtt{X}}(\zeta_m)$ then the resulting outward electrical current is

$$I_{\mathtt{X}}(\zeta_m) = -z_{\mathtt{X}}\mathcal{F}\mathcal{S}P_{\mathtt{X}}(\zeta_m)\Psi\left(z_{\mathtt{X}}\zeta_m\right)\left([\mathtt{X}]_{\text{out}} - [\mathtt{X}]\, e^{z_{\mathtt{X}}\zeta_m}\right) \tag{3}$$

with $\Psi(u) = u\left(e^u - 1\right)^{-1}$. From the n measures for each species represented on Figure S1, we perform a general least square fit by using

$$\chi^2_{\mathtt{X}} = \sum_{i=1}^{n}\left[I(\zeta_i) + z_{\mathtt{X}}\mathcal{F}\mathcal{S}P_{\mathtt{X}}(\zeta_i)\Psi\left(z_{\mathtt{X}}\zeta_i\right)\left([\mathtt{X}]_{\text{out}} - [\mathtt{X}]\, e^{z_{\mathtt{X}}\zeta_i}\right)\right]^2 . \tag{4}$$

Within the range of observed intensities, we experimentally found that the permeabilities match quite satisfactorily the form

$$\mathcal{S}P_{\mathtt{X}}(\zeta_m) = \tilde{A}_{\mathtt{X}}e^{\tilde{B}_{\mathtt{X}}\zeta_m} + \tilde{C}_{\mathtt{X}} \tag{5}$$

with $\tilde{C}_{\mathtt{Na}^+} = \tilde{C}_{\mathtt{Cl}^-} = 0$ and $\tilde{C}_{\mathtt{K}^+} \neq 0$ due to the greater variability of $I_{\mathtt{K}^+}$. The minimization of $\chi^2_{\mathtt{X}}$ with respect to the corresponding coefficients provides the following coefficients and their standard error, with a correlation coefficient greater than 0.998:

$$\begin{aligned}
\mathcal{S}P_{\mathtt{K}^+}(\zeta_m)/(\mu\text{m}^3.\text{s}^{-1}) &= (3.70 \pm 0.70)\, e^{(0.43\pm0.05)\,\zeta_m} + (1.5 \pm 0.5) \\
\mathcal{S}P_{\mathtt{Na}^+}(\zeta_m)/(\mu\text{m}^3.\text{s}^{-1}) &= (1.36 \pm 0.03)\, e^{(0.05\pm0.01)\zeta_m} \\
\mathcal{S}P_{\mathtt{Cl}^-}(\zeta_m)/(\mu\text{m}^3.\text{s}^{-1}) &= (1.19 \pm 0.02)\, e^{(0.06\pm0.01))\zeta_m}
\end{aligned} \tag{6}$$

and the corresponding fitted intensities are shown on Figure S1. The permeabilities found here are compatible with values found in other studies [6, 7].

Methods

Obtaining an analytical expression of the pH dynamics

As seen in the manuscript, let us assume that the protic subsystem is defined by only five species (by neglecting $\mathtt{CO}_3^{2-}$), namely

$$\overrightarrow{[\mathtt{X}]} = \begin{bmatrix} [\mathtt{H}^+] \\ [\mathtt{HO}^-] \\ [\mathtt{HCO}_3^-] \\ [\mathtt{HY}] \\ [\mathtt{Y}^-] \end{bmatrix}. \tag{7}$$

We have three equilibria leading to

$$\vec{\Gamma} = \begin{bmatrix} K_w - [\mathtt{H}^+][\mathtt{HO}^-] \\ K_1'\Pi_{\mathtt{CO}_2} - [\mathtt{H}^+][\mathtt{HO}^-] \\ K_\mathtt{Y}[\mathtt{HY}] - [\mathtt{H}^+][\mathtt{Y}^-] \end{bmatrix},\ \partial_t\vec{\Gamma} = \begin{bmatrix} 0 \\ K_1'\partial_t\Pi_{\mathtt{CO}_2} \\ 0 \end{bmatrix} \tag{8}$$

with a stoichiometric coefficients matrix

$$\boldsymbol{\nu} = \begin{bmatrix} 1 & 1 & 0 & 0 & 0 \\ 1 & 0 & 1 & 0 & 0 \\ 1 & 0 & 0 & -1 & 1 \end{bmatrix}. \tag{9}$$

The following formal computation can be perform manually or using a symbolic mathematical software like `maxima` (see http://maxima.sourceforge.net) to get

$$\boldsymbol{\Phi} = \begin{bmatrix} -[\mathtt{HO}^-] & -[\mathtt{H}^+] & 0 & 0 & 0 \\ -[\mathtt{HCO}_3^-] & 0 & -[\mathtt{H}^+] & 0 & 0 \\ -[\mathtt{Y}] & 0 & 0 & K_\mathtt{Y} & -[\mathtt{H}^+] \end{bmatrix}. \tag{10}$$

The relaxed variations of concentrations writes

$$\partial_t\overrightarrow{[\mathtt{X}]} = \left(\boldsymbol{I}_5 - \boldsymbol{\nu}^\intercal\left(\boldsymbol{\Phi}\boldsymbol{\nu}^\intercal\right)^{-1}\boldsymbol{\Phi}\right)\begin{bmatrix} -\rho_{\mathrm{NHE}} \\ 0 \\ -\rho_{\mathrm{AE}} \\ 0 \\ 0 \end{bmatrix} - \boldsymbol{\nu}^\intercal\left(\boldsymbol{\Phi}\boldsymbol{\nu}^\intercal\right)^{-1}\partial_t\vec{\Gamma} \tag{11}$$

from which the variation of $[\mathtt{H}^+]$ can be extracted once the other protic concentrations are substituted by their equilibrium values

$$\partial_t[\mathtt{H}^+] = \Theta\left([\mathtt{H}^+], \mathtt{Y}_0\right)\left(\left(\rho_{\mathrm{AE}} - \rho_{\mathrm{NHE}}\right) + \frac{K_1'}{[\mathtt{H}^+]}\partial_t\Pi_{\mathtt{CO}_2}\right) \tag{12}$$

with

$$\Theta\left([\mathtt{H}^+], \mathtt{Y}_0\right) = \frac{[\mathtt{H}^+]^2}{[\mathtt{H}^+]^2 + K_1'\Pi_{\mathtt{CO}_2} + K_w + \mathtt{Y}_0 K_\mathtt{Y}\left(\frac{[\mathtt{H}^+]}{[\mathtt{H}^+] + K_\mathtt{Y}}\right)^2}. \tag{13}$$

If the enzymatic rate can be expressed from $[\mathtt{H}^+]$ only, then the equation (12) is sufficient to simulate the exact evolution of $[\mathtt{H}^+]$. In that case, a further formal work shall similarly provide a single differential

equation which would take into account CO_3^{2-} or other protic species (for example lactate and lactic acid). Moreover, the chemical damping Θ can be factorized into

$$\Theta\left([H^+], Y_0\right) = \frac{[H^+]^2}{[H^+]^2 + K_1'\Pi_{CO_2} + K_w}\frac{1}{1+\beta_Y Y_0} \tag{14}$$

with

$$\beta_Y = \frac{K_Y}{[H^+]^2 + K_1'\Pi_{CO_2} + K_w}\left(\frac{[H^+]}{[H^+] + K_Y}\right)^2. \tag{15}$$

This factor, as described in the manuscript, is a kinetic buffer effect: the presence of the buffer doesn't affect the stationary pH, but damps the pH variations.

Natural Overshoot

Pertubative framework

From the algebraic analysis of the protic chemical system, we can show that the evolution of the proton concentration h, relatively to the steady state concentration $h^\star$, is always of the form

$$\partial_t h = F(h - h^\star) + Q(h)G(t). \tag{16}$$

We have the following properties.

- F is a control function imposed by the enzymatic regulation system. We assume that $F(x)$ has a unique root in 0, such that $F(0) = 0$. In addition, this steady point is assumed to be linearly stable: $F'(0) < 0$.
- G is an external perturbation function, so that its integral from the beginning of the perturbation to the end of the perturbation is zero, and its value at the end of the perturbation is zero as well: it can be seen as the quantity of protons that are "injected" by the experimenter then "removed" in the same quantity by the cell.
- Q is a chemical dampening function which characterizes the protic system. Physically, we have $Q > 0$ or $Q < 0$ but never $Q = 0$, which would mean that the system can both amplify and counteract the perturbation.

Conservation Laws

Let us make a simple perturbation starting from $h^\star$ at t_0, going to h_1 at t_1 and coming back to $h^\star$ at t_2, with the possible situation where $t_2 \to +\infty$. So far, we do not make any assumption about the form of G but at the end of the perturbation we have

$$\int_{t_0}^{t_2} G(u)du = 0. \tag{17}$$

and we compute

$$\int_{t_0}^{t_1} G(u)du = \Gamma = -\int_{t_1}^{t_2} G(u)du. \tag{18}$$

Since $Q \neq 0$, we can rewrite (16) as

$$\frac{\partial_t h}{Q(h)} = \frac{F(h - h^\star)}{Q(h)} + G(t) \tag{19}$$

and integrate it on the two time ranges. From t_0 to t_1 we obtain

$$\int_{t_0}^{t_1} \frac{\partial_t h}{Q(h)}\, dt = \int_{h^\star}^{h_1} \frac{dh}{Q(h)} = \Lambda \tag{20}$$

and finally

$$\Lambda = \int_{t_0}^{t_1} \frac{F(h - h^\star)}{Q(h)}\, dt + \Gamma. \tag{21}$$

Similarly from t_1 to t_2 we obtain

$$\int_{h_1}^{h^\star} \frac{dh}{Q(h)} = -\Lambda = \int_{t_1}^{t_2} \frac{F(h - h^\star)}{Q(h)}\, dt - \Gamma \tag{22}$$

so we always have

$$\int_{t_0}^{t_1} \frac{F(h - h^\star)}{Q(h)}\, dt + \int_{t_1}^{t_2} \frac{F(h - h^\star)}{Q(h)}\, dt = 0 \tag{23}$$

Answer to a simple perturbation

We make the following perturbation

$$\begin{cases} G(t_0 \to t_1) & > & 0 \\ G(t_1 \to t_2) & < & 0 \end{cases} \tag{24}$$

respecting the condition (17). Let us assume that $Q > 0$ since we can change the sign of Q and G to produce the same result. The integration of (16) shows that h is increasing from $h^\star$ to h_1 between t_0 and t_1. Accordingly, the first integral of the relation (23) is negative since, by assumption, $F\left((h - h^\star) > 0\right) < 0$. Consequently, the second integral of the relation (23) must be positive. This is possible only if, somewhere between t_1 and t_2, we have $F > 0$. In other words, somewhere between t_1 and t_2, we have $h < h^\star$: the pH returns to the steady state value by overshooting, which is what is observed experimentally.
All this demonstration holds if we change the sign of G or Q, which will simply change the sign of $h - h^\star$.

Parametrized perturbation

In the extended specific case where ϕ represents an external intensive perturbation, $\phi' = \partial_t \phi$ and the variation of the proton concentration h is

$$\partial_t h = F(h - h^\star(\phi(t))) + Q(h)\phi'(t), \tag{25}$$

we obtain

$$\int_{t_0}^{t_1} \frac{F(h - h^\star(\phi(t))}{Q(h)}\, dt + \int_{t_1}^{t_2} \frac{F(h - h^\star(\phi(t)))}{Q(h)}\, dt = 0 \tag{26}$$

and the previous proof is still valid if $h^\star$ is a non decreasing function of ϕ.

Results

Simulation

We carried out the simulation of a Π_{CO_2} step with the same rise than in the Π_{CO_2} spike of the article: the pressure is increased from 40 mmHg to 48 mmHg in 1 minute. We use the initial values reported in the Datasets. We observe the relaxation to a new steady state pH without overshoot. This result is perfectly coherent with the predicted steady state depicted on Figure 2 (in the manuscript), when Π_{CO_2} is modified while the ratio $V_{\mathrm{AE}}/V_{\mathrm{NHE}}$ remains unchanged.

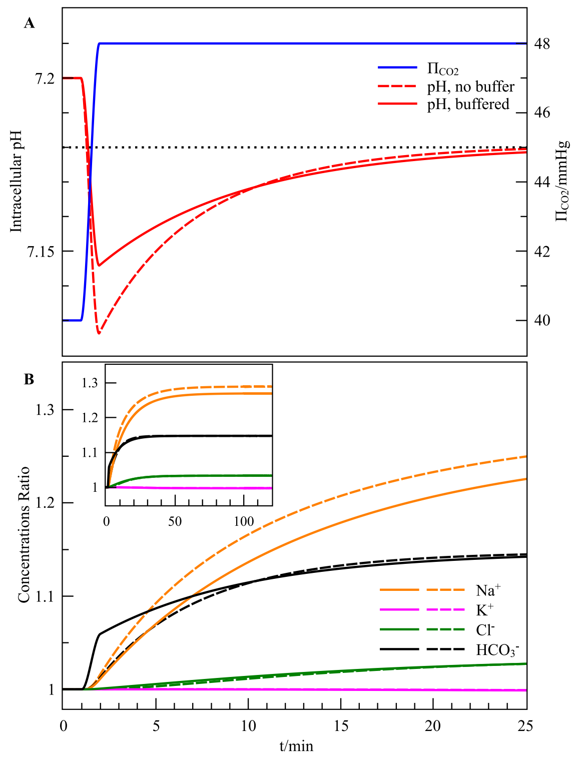

Figure S2. Forced acidosis by a simulated Π_{CO_2} step. (A) The pH returns to its predicted value,without buffer or with a 60 mM buffer (dashed line). (B) The ionic ratios relative to the initial values are reported as well, and are converging to some different steady state values (see inset).

Computing enzymatic constants from steady state values

The equations to be solved to describe the steady state are the following (see the article for the notations, the exponent $\star$ marks the resting values):

$$\left\{\begin{array}{lcl} 0 & = & \lambda^\star_{\texttt{K}^+} + \lambda^\star_{\texttt{Na}^+} - \lambda^\star_{\texttt{Cl}^-} - \rho^\star_{\texttt{NaK}} \\ 0 & = & \lambda^\star_{\texttt{K}^+} + 2\rho^\star_{\texttt{NaK}} \\ 0 & = & \lambda^\star_{\texttt{Na}^+} - 3\rho^\star_{\texttt{NaK}} + \rho^\star_{\mathrm{NHE}} \\ 0 & = & \lambda^\star_{\texttt{Cl}^-} + \rho^\star_{\mathrm{AE}} \\ 0 & = & \rho^\star_{\mathrm{NHE}} - \rho^\star_{\mathrm{AE}} \end{array}\right. \tag{27}$$

where each λ terms depends on the membrane potential E_m and on the concentrations, and where each ρ term is associated to an enzymatic catalytic constant and depends on the concentrations as well. As mentioned in the article, this system is under-determined since the sum of the first three equations minus the sum of the two last ones is zero. Yet, assuming we know the steady states concentrations, we show how to compute the enzymatic parameters.
Before proceeding further, we obtain the electric equation

$$0 = \frac{3}{2}\lambda^\star_{\texttt{K}^+} + \lambda^\star_{\texttt{Na}^+} - \lambda^\star_{\texttt{Cl}^-}. \tag{28}$$

If the permeabilites $P_{\texttt{K}^+}(E_m)$, $P_{\texttt{Na}^+}(E_m)$ and $P_{\texttt{Cl}^-}(E_m)$ are experimentally measured (see Datasets), then there exists only one resting potential $E^\star_m$. Because of the uncertainty in the experimental measures, $E^\star_m$ can be tuned by multiplying $P_{\texttt{K}^+}$ (the highest permeability) by a small constant positive coefficient. From now on, all the $\lambda^\star$ terms and $E^\star_m$ are determined.
In particular, we deduce

$$\rho^\star_{\texttt{NaK}} = -\frac{1}{2}\lambda^\star_{\texttt{K}^+} \tag{29}$$

and we assess the value of $V_{\texttt{NaK}}$, the cellular maximum $\texttt{Na}^+/\texttt{K}^+$ exchange rate.
Similarly, we note that

$$\rho^\star_{\mathrm{AE}} = -\lambda^\star_{\texttt{Cl}^-} \tag{30}$$

and we deduce the value of V_{AE}, the cellular maximum $\texttt{Cl}^-/\texttt{HCO}_3^-$ exchange rate.
Finally, since we know $[\texttt{H}^+]^\star$ and that we have $\rho^\star_{\mathrm{NHE}} = \rho^\star_{\mathrm{AE}}$, we also compute V_{NHE}, the cellular maximum $\texttt{H}^+/\texttt{Na}^+$ exchange rate.

Experimental Adequacy

Figure S3 shows a typical pH recording in *Helix* snail neurons [8] submitted to a $\texttt{CO}_2$ pulse (Figure 5A of the original article). According to the authors' experimental setup, we use the parameters reported in Table S3.

Parameter	Intracellular Value	Extracellular Value
$\mathrm{pH}^\star$	7.35	7.0
$E^\star_m$	–55 mV	–
$[\texttt{K}^+]^\star$	140 mM	4 mM
$[\texttt{Na}^+]^\star$	10 mM	140 mM
$[\texttt{Cl}^-]^\star$	20 mM	100 mM

Table S3. Steady-state ionic atmospheres. These values correspond to the *Helix* snail neurons.

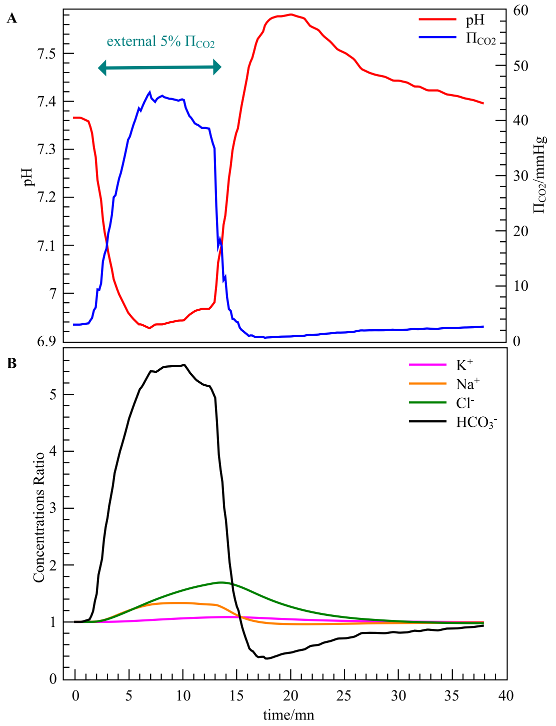

Figure S3. pH overshoot in *Helix* snail neurons. (A) Experimental pH overshoot (red line) following an external $\Pi_{\mathtt{CO}_2}$ exposure. The internal $\Pi_{\mathtt{CO}_2}$ increase is computed (blue line) (B) Corresponding computed ionic ratios, relative to the initial values.

At first, the neuron is exposed to an atmosphere expunged from $\mathtt{CO}_2$, so that $\Pi_{\mathtt{CO}_2}$ is minimal within the neuron. To maintain an internal pH of 7.35 with a low carbon dioxide pressure, the neuron has a ratio $V_{\mathrm{AE}}/V_{\mathrm{NHE}} \simeq 0.12$ (expected from Figure 2), which confers the cell a high sensitivity to $\Pi_{\mathtt{CO}_2}$.

Then a 5% $\mathtt{CO}_2$ ($\Pi_{\mathtt{CO}_2} \simeq$ 38mmHg) is imposed on the cell for a few minutes, before returning to the initial state.

The model can be used to reconstruct the $\mathtt{CO}_2$ pressure build-up within the neuron. We reported the measured pH on Figure S3A (red line). With an estimated internal $\Pi_{\mathtt{CO}_2}$ of about 3 mmHg, wer recursively compute a piecewise affine $\Pi_{\mathtt{CO}_2}$ function by matching the computed pH with the experimental pH. The corresponding $\Pi_{\mathtt{CO}_2}$, *without any modification of the model*, and is reported on Figure S3A (blue line). Remarkably, we observe that the computed pressure matches a 5% $\Pi_{\mathtt{CO}_2}$ jump imposed by the experiment: considering the experimental uncertainties in the pH curve, the computed $\Pi_{\mathtt{CO}_2}$ function is quite satisfactory.

The bicarbonate level in the cell is highly modified (see Figure S3B, black line). Concurrently (as show on Figure S3B, green line) an important $\mathtt{Cl}^-$ intake occurs (since AE2 is running at high speed), but it is counterbalanced by an almost equivalent $\mathtt{Na}^+$ entrance (Figure S3B, orange line). Finally, a slight $\mathtt{K}^+$ input (Figure S3B, pink line) adds up to produce a not significant 1 mV hyperpolarization during the whole process.

Interestingly enough, our model offers the possibility to guess some unknown data or functions from an experimental result, and can be used to reconstruct the $\Pi_{\mathtt{CO}_2}$ build-up during pH changes in any cellular system.

References

1. Lovosilo D, Alloatti G, Bonelli G, Tessitore L, Baccino FM (1988) Potassium and calcium currents and action potentials in mouse Balb/c 3T3 fibroblasts. European J Physiol 412: 530-534.

2. Lacroix J, Poët M, Maehrel C, Counillon L (2004) A mechanism for the activation of the Na^+/H^+ exchanger NHE-1 by cytoplasmic acidification and mitogens. EMBO reports 5: 91-96.

3. Gadsby DC, Nakao M (1989) Steady-state current-voltage relationship of the Na/K pump in guinea pig ventricular myocytes. J Gen Physiol 94: 511-537.

4. Despa S, Bers DM (2003) Na^+/K^+ pump current and $[Na]_i$ in rabbit ventricular myocytes: Local $[Na]_i$ depletion and Na^+ buffering. Biophysical Journal 84: 4157-4166.

5. Hille B (2001) Ion channels of excitable membranes. Sinauer Associates Inc. Publishers.

6. Hunter MJ (1977) Human erythocite anion permeabilities measured under conditions of net charge transfer. J Physiol 268: 35-59.

7. Strickhol A (1981) Ionic permeability of K^+, Na^+ and Cl^- in potassium depolarized nerve. Biophys J 35: 677-697.

8. Roos A, Boron WF (1981) Intracellular pH. Physiol Rev 61: 296-434.
